# Physical Rehabilitation Using Oromotor Stimulation, Manual Airway Clearance Technique, Positioning, and Tactile and Kinaesthetic Stimulation (PROMPT) Protocol in Low-Birth-Weight Triplets With Neonatal Respiratory Distress: A Case Series

**DOI:** 10.7759/cureus.67605

**Published:** 2024-08-23

**Authors:** Sakshi Desai, HV Sharath, Gurjeet Kaur, Moh'd Irshad Qureshi

**Affiliations:** 1 Department of Paediatric Physiotherapy, Ravi Nair Physiotherapy College, Datta Meghe Institute of Higher Education and Research, Wardha, IND; 2 Center for Advanced Physiotherapy Education and Research (CAPER), Ravi Nair Physiotherapy College, Datta Meghe Institute of Higher Education and Research, Wardha, IND; 3 Department of Neuro-Physiotherapy, Ravi Nair Physiotherapy College, Datta Meghe Institute of Higher Education and Research, Wardha, IND

**Keywords:** low birth weight, physiotherapy, triplets, prompt protocol, neonatal respiratory distress syndrome(nrds)

## Abstract

Neonatal respiratory distress syndrome (RDS), a severe respiratory illness that is likely to affect preterm newborns especially those who were born preterm with low birth weight (LBW) or multiple births, is one of the complications that preterm babies are likely to develop. Physical Rehabilitation using Oromotor Stimulation, Manual Airway Clearance Technique, Positioning, and Tactile and Kinaesthetic Stimulations (PROMPT) is the intervention followed in this study to determine its effectiveness in the treatment of RDS in LBW triplets. The PROMPT protocol involves interventions such as manually promoting the airway, positioning, oral motor stimulation, and tactile and kinesthetic stimulation. The study examined triplets of similar weight, 1.23g, 1.36g, and 1.18g, at birth. Thus, all known triplets were suffering from the symptoms of RDS like fast breathing and grunting. They were born via premature delivery at 30+5 weeks of pregnancy. Chest X-rays were used as a diagnostic tool for assessing RDS. At the same time, the PROMPT protocol was administered and significant improvements were seen in respiratory health and there was reduced use of mechanical ventilation. The PROMPT protocol shows how effectively an organized method can be applied to treat RDS in LBW triplets.

## Introduction

Neonatal respiratory distress syndrome (NRDS) in preterm neonates is mainly caused by a deficiency in surfactant. This deficiency results in increased surface tension in the alveoli and small airways, thereby reducing lung compliance [1,2]. As per LaPlace's law (P=2T/R), the pressure required to keep alveoli open increases with surface tension. A lack of surfactant leads to widespread atelectasis, which reduces gas exchange, damages the respiratory epithelium, triggers inflammation, and causes pulmonary edema. Mechanical ventilation, often required to treat RDS, can exacerbate lung damage by increasing oxidative stress and causing over-distension, which further deactivates surfactants. This condition leads to lactic acidemia, as well as hypoxemia and tissue damage [3-5].

The neonates with RDS are born prematurely and present with symptoms of respiratory problems soon after birth or during the first hours and days of life. Some signs that may be observed in such neonates include reduced breath sounds on auscultation and possibly peripheral pulses [6,7]. Some clinical signs in neonates with RDS include increased respiratory rate, grunting, nasal flaring, sternal retractions, cyanosis, poor peripheral circulation, and reduced breath sounds throughout the chest. If this condition is left untreated, the condition usually worsens over time, which can be in less than 48-72 hours, leading to respiratory complications and lethargy [8-10].

It is commonly recognized that multiple pregnancies, particularly triplet pregnancies, typically result in preterm delivery. This is mostly due to mechanical considerations, such as the uterus's capacity to hold the fetus [11]. NRDS is more likely to occur under certain conditions. The primary cause of NRDS in preterm babies is lung immaturity brought on by insufficient surfactant production [12]. Furthermore, triplet birth weights are frequently significantly lower than singlet birth weights, which increases the risk due to the association between low birth weight and undeveloped organs, particularly the lungs. Multiple pregnancies are associated with a higher risk of problems, such as early membrane rupture, preterm labor, and premature birth [13,14]. In the above case report, we provide a systematically organized Physical Rehabilitation Using Oromotor Stimulation, Manual Airway Clearance Technique, Positioning, and Tactile and Kinaesthetic Stimulation (PROMPT) protocol for neonates diagnosed with NRDS. The implementation of the PROMPT protocol in neonates with NRDS was shown to be an effective intervention.

## Case presentation

Prenatal history

A 36-year-old female conceived via in vitro fertilization and underwent routine prenatal care. She had a weight gain of 11 kg during gestation and had a previous history of attempted abortion. Her nutritional intake was adequate and there was a presence of fetal movements. For the past year, she has been taking thyroxine 25 mg daily for the treatment of hypothyroidism. Ultrasound findings are provided in Table 1.

**Table 1:** Ultrasound findings FA, Fetus A; FB, fetus B;  FC, fetus C; OS: orifice of cervix; DVP, deepest vertical pocket

Report Date	Day 1	Day 15	Day 30	Day 45	Day 60
Fetus A	Triamniotic trichorionic triplets with intrauterine live fetus FA (away from OS) corresponding to average gestational age of 14 weeks and four days and effective fetal weight of 102 g.	Triamniotic trichorionic triplets with intrauterine live fetus FA (away from OS) corresponding to average gestational age of 18 weeks and five days and effective fetal weight of 268 g.	Triamniotic trichorionic fetus (FA) of average gestational age of 21 weeks and two days and corresponding to weight of 427 g	Triamniotic trichorionic triplets with intrauterine live fetus FA (near the OS) corresponding to the average gestational age of 26 weeks and two days and effective fetal weight of 896 g; DVP: 5.4 cm	Triamniotic trichorionic fetus FA (near OS) of average gestational age of 30 weeks and one day and corresponding to a fetal weight of 1516 g
Fetus B	Triamniotic trichorionic triplets with intrauterine live fetus FB (away from OS) corresponding to average gestational age of 14 weeks and six days and effective fetal weight of 112 g.	Triamniotic trichorionic triplets with intrauterine live fetus FB (maternal left) corresponding to average gestational age of 19 weeks and one day and effective fetal weight of 287 g.	Triamniotic trichorionic fetus (FB) of average gestational age of 21 weeks and two days and corresponding to weight of 419 g	Triamniotic trichorionic triplets with intrauterine live fetus FB (maternal right) corresponding to the average gestational age of 25 weeks and five days and effective fetal weight of 835 g; DVP: 4.1 cm	Triamniotic trichorionic fetus FB (maternal right) of average gestational age of 30 weeks and two days and corresponding to a fetal weight of 1464 g
Fetus C	Triamniotic trichorionic triplets with intrauterine live fetus FC (maternal right) corresponding to an average gestational age of 18 weeks and five days and effective fetal weight of 266 g. Cervical canal length: 3.8 cm.	Triamniotic trichorionic triplets with intrauterine live fetus FC (away from OS) corresponding to an average gestational age of 17 weeks and one day and effective fetal weight of 177 g.	Triamniotic trichorionic fetus (FC) of average gestational age of 24 weeks and one day and corresponding to a fetal weight of 657 g	Triamniotic trichorionic triplets with intrauterine live fetus FC (maternal left) corresponding to average gestational age of 27 weeks and effective fetal weight of 1003 g. DVP: 6.2 cm	Triamniotic trichorionic fetus FC (maternal left) of average gestational age of 30 weeks and four days and corresponding to a weight of 1547 g

Natal history

Baby A

A newborn male baby of first birth order with a birth weight of 1. 23 kg was delivered through a lower abdominal cesarean section to a G2A1 mother, at 30+5 weeks gestation due to triplet pregnancy and preterm premature rupture of the membrane. The neonate cried immediately at the time of birth; the Apgar score, assessed for 1 minute, was 4/10; for 5 minutes, it was 7/10. As the baby was born with a very low birth weight and respiratory issues, he was admitted to the neonatal intensive care unit (NICU).

Baby B

A newborn female baby of second birth order with a birth weight of 1.36 kg was delivered through a lower abdominal cesarean section to a G2A1 mother, at 30+5 weeks gestation due to triplet pregnancy and preterm premature rupture of the membrane. The neonate did not cry immediately at the time of birth; the Apgar score, assessed for 1 minute, was 4/10; for 5 minutes, it was 8/10. As the baby was born with a very low birth weight and respiratory issues, she was admitted to the NICU.

Baby C

A newborn male baby of third birth order with a birth weight of 1.6 kg was delivered through a lower abdominal cesarean section to a G2A1 mother, at 30+5 weeks gestation due to triplet pregnancy and preterm premature rupture of membrane. The neonate did not cry immediately at the time of birth; the Apgar score, assessed for 1 minute, was 6/10; for 5 minutes, it was 8/10. As the neonate was born with a very low birth weight and respiratory issues, he was admitted to the NICU for further medical management.

Postnatal history

The neonates exhibited signs of respiratory distress, including sternal retractions and reliance on accessory muscles for breathing. Following a chest X-ray, the newborns were diagnosed with NRDS.

On examination

The physiological parameters of Baby A, Baby B, and Baby C showed the subsequent measurements. Baby A displayed a pulse rate of 170 bpm, a respiratory rate of 58 beats/minute, and an oxygen saturation level (SpO_2_) of 98%. Baby B's pulse rate appeared to be at 166 bpm, along with a respiratory rate of 53 b/m and a SpO_2_ of 96%. Lastly, Baby C revealed a pulse rate of 160 beats per minute, a respiratory rate of 56b/m, and SpO_2_ of 97% (Table 2).

**Table 2:** Vital signs of Baby A, Baby B, and Baby C

Vitals	Baby A	Baby B	Baby C
Pulse rate	170bpm	166bpm	160bpm
Respiratory rate	58b/m	53b/m	56b/m
SpO_2_	98%	96%	97%

The anthropometric measurements performed on three preterm neonates revealed variations in their physical appearance upon delivery. Baby A weighed 1.23 kg, had a head circumference of 28.5 cm, a chest circumference of 27.5 cm, and a length of 44 cm. In contrast, Baby B weighed 1.36 kg, had a head circumference of 29 cm, a chest circumference of 28 cm, and a length identical to Baby A, measuring 44 cm. Baby C, the smallest among the triplets, exhibited a birth weight of 1.18 kg, a head circumference of 27.5 cm, a chest circumference of 26.5 cm, and length of 42 cm (Table 3).

**Table 3:** Birth weight, head circumference, chest circumference, and length measurements of the three newborns

Anthropometric measurements	Baby A	Baby B	Baby C
Birth weight	1.23 kg	1.36 kg	1.18 kg
Head circumference	28.5 cm	29 cm	27.5 cm
Chest circumference	27.5 cm	28 cm	26.5 cm
Length	44 cm	44 cm	42 cm

During the examination of cardiorespiratory function, Baby A demonstrated an abdominothoracic breathing pattern characterized by an elevated respiratory rate and symmetrical chest movement. However, the neonates showed prolonged inspiratory phases, increased respiratory effort, and the use of accessory muscles, indicating increased respiratory workload. In comparison, Baby B also displayed an abdominothoracic breathing pattern but with mild tachypnea and asymmetrical chest movement. Similar to Baby A, Baby B showed extended inspiratory phases, heightened respiratory effort, and the utilization of accessory muscles. Baby C exhibited an abdominothoracic breathing pattern with an increased respiratory rate, symmetrical chest movement, prolonged inspiratory phases, increased respiratory (Table 4).

**Table 4:** Cardiorespiratory examination in Baby A, Baby B, and Baby C The table depicts the type of breathing, chest symmetry and inspiratory:expiratory ratio in Baby A, Baby B, and Baby C

Cardiorespiratory Examination	Baby A	Baby B	Baby C
Type of breathing	Abdominothoracic with increased respiratory rate	Abdominothoracic with mild tachypnea	Abdominothoracic with increased respiratory rate
Chest symmetry	Bilaterally symmetrical chest	Asymmetrical chest	Bilaterally symmetrical chest
Inspiratory:expiratory ratio	Extended inspiratory phase with increased respiratory effort	Extended inspiratory phase with increased respiratory effort	Extended inspiratory phase with increased respiratory effort

Radiological examination 

**Figure 1:**
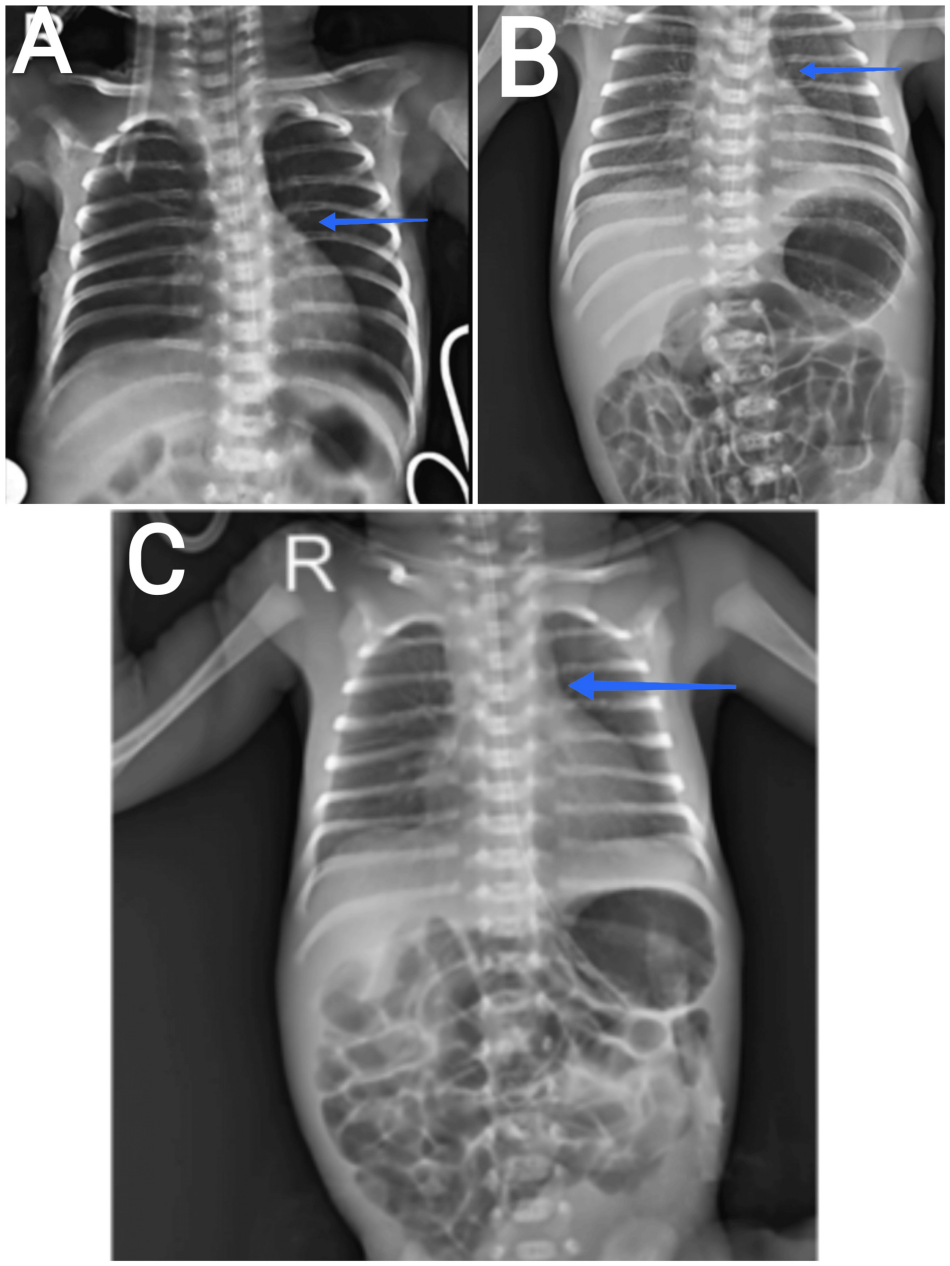
Chest radiograph of Baby A, Baby B, and Baby C The blue arrow in Figure 1A shows the decreased bronchovascular markings; Figure 1B and Figure 1C show increased bronchovascular markings

Chest X-rays were taken for Baby A, Baby B, and Baby C, all in the posterior-anterior view (PA) view and during inspiration. The trachea was observed to be central in all three cases. For Baby A, the lung fields showed decreased bronchovascular markings, indicating a possible reduction in pulmonary blood flow or air trapping. The diaphragm appeared normal, with the right dome slightly elevated compared to the left. Both the cardiophrenic and costophrenic angles were clear, suggesting no significant fluid accumulation or other abnormalities in these regions. In the case of Baby B, the lung fields exhibited increased bronchovascular markings, which could indicate increased blood flow or congestion. The right and left domes of the diaphragm were at the same level, showing no elevation on either side. However, the cardiophrenic angle was obliterated, possibly suggesting the presence of a pathology like a mass or lymphadenopathy. The costophrenic angles were clear, indicating no pleural effusion or other fluid-related issues. Baby C's lung fields also displayed increased bronchovascular markings, similar to Baby B, which could indicate similar underlying conditions. The diaphragm appeared normal, with the right dome slightly elevated, as observed in Baby A. Both the cardiophrenic and costophrenic angles were clear, showing no signs of fluid accumulation or other abnormalities in these areas (Figure 1).

Intervention 

The subsequent course of the neonate hospitalization and response to the PROMPT protocol are discussed in Table 5 and treatment was continued up to the discharge of the neonates from NICU [15,16].

**Table 5:** Physical Rehabilitation using Oromotor stimulation, Manual airway clearance technique, Positioning, and Tactile and kinaesthetic stimulation (PROMPT) protocol

Intervention		Procedure	Duration
Oromotor stimulation	Cheek stretch	Using your index finger, gently press on the tissue at the nasal bridge. Using your finger, make a C shape and slide it down to the lip's corner after initially going toward the ear. On the opposite side, carry out the same procedure.	2 min
	Upper lip stimulation	To compress the tissue, lightly push the index finger on the top lip's corner. Your finger should go from one corner to the center and back again in a circular pattern. To finish the movement, reverse the direction.	2 min
	Lower lip stimulation	To compress the skin, lightly press your index finger on the lower lip's corner. Make a circular movement with your finger, beginning at the corner, moving to the middle, and ending at the opposite corner.	2 min
	Upper gum stimulation	By starting your finger in the middle of the gum, moving it to the rear of the mouth, and then back to the center, you may gradually apply strong pressure.	2 min
	Lower gum stimulation	Place your finger in the middle of the gum, push firmly and steadily, and then gently slide it in the direction of the rear of your mouth.	2 min
	Temporomandibular joint stroking	These techniques involve focusing pressure on the surrounding muscles, kneading the jaw joint softly, and moving in circles. Enhancing orofacial muscular coordination, reducing muscle tension, and increasing blood flow are the goals.	2 min
Airway clearance technique	Percussion	To create vibrations in the lung airways, chest percussion entails tapping the chest using percussor cups, which resemble suction cups. By loosening mucus, these vibrations facilitate better cough expulsion.	30 percussions for five sets
	Gentle vibrations	During exhale, a short, delicate trill-like motion was used to delicately impart vibrations. After percussion, gentle vibrations were applied to help move secretions toward the bigger airways. The targeted location on the chest wall was covered with the fingers of one hand to manually apply the chest vibrations to each infant. The hand and forearm muscles contracted isometrically to produce a faint vibrating motion. Throughout the procedure, the infant's head was supported by the other hand, which was held with the palm cupped to cradle it.	30 vibrations for five sets
	Postural drainage	During the draining of the anterior parts of the left and right upper lobes, the infant was placed flat on the back. The neonate was tilted forward at a 90-degree angle to allow for draining the left and right lateral basal portions of the lower lobes. After that, the top regions of the lower ribs were percussed. Furthermore, the sides of the chest below the clavicles were pounded, extending into the nipple region, with caution to prevent direct pressure on the sternum.	Each drainage posture was maintained for 5 min per position.
Positioning	Swaddling	It is traditional to swaddle newborns, limiting their movement by enveloping them securely in a blanket or piece of fabric. It's a typical technique to help newborns feel safe and sleep better. To reduce hazards, it's essential to swaddle safely though. The following are some fundamental ideas about the swaddling position: reverse placement permission at the hips; put the infant's hands up or face down; skew the legs.	3 hours per day
Tactile stimulations	-	The baby was stimulated tactilely by being softly caressed with moderate pressure, which required the use of both hands.	Each stimulation treatment was given for 10 min, twice per day at least after 2 hours post-feeding
Kinesthetic stimulations	-	Passive movements with mild joint compression were administered to provide kinesthetic stimulation to the bilateral upper and lower extremity.	Each stimulation treatment was given for 10 min, twice per day at least after 2 hours post-feeding

Outcome measures 

Silverman-Anderson Respiratory Severity Score (RSS) Questionnaire 

The Silverman-Anderson score is a clinical instrument that assesses respiratory distress in neonates using five parameters: nasal flaring, grunting, intercostal retractions, expiratory grunting, and chest wall movement. Each parameter is assigned a value between 0 and 2, with higher scores indicating more discomfort. The total score assists doctors in determining the urgency of intervention, such as oxygen therapy or breathing support, as well as tracking patient response to treatment. While the score is useful for detecting respiratory distress, it is subjective and does not address the underlying causes of respiratory problems.

Oromotor Assessment Scale (OMAS)

The Oromotor Assessment Scale (OMAS) is a clinical measure used to assess the function and coordination of oral motor abilities in patients, particularly having feeding difficulties. The scale evaluates a variety of factors such as lip closure, tongue movement, and oral transit time. It gives vital information on the integrity and efficiency of orofacial motor abilities. This measure is especially useful in identifying and planning therapies. By grading each parameter methodologically, healthcare providers design their therapy approaches to improve oral motor skills and thus improve quality of life (Table 6).

**Table 6:** Outcome Measures Silverman-Anderson Respiratory Severity Score (RSS) Questionnaire: A total score of 0 suggests no distress; a score of 1-4 mild respiratory distress (RD); a score of 5-7 moderate RD; a score of >7 severe distress or impending respiratory failure. Oral Motor Assessment Scale: Scores of 0-6 indicate severe impairment; 7-12 is moderate impairment; 13-18 is mild impairment; 19-21 is minimal or no impairment. PROMPT: Physical Rehabilitation using Oromotor stimulation, Manual airway clearance technique, Positioning, and Tactile and kinaesthetic stimulation

Length of hospital stay	Baby A		Baby B		Baby C	
	38 days		30 days		28 days	
Outcome measures	Pre-administration of PROMPT protocol			Post-administration of PROMPT protocol		
	Baby A	Baby B	Baby C	Baby A	Baby B	Baby C
SPO_2_ (%)	78	75	73	95	96	98
Silverman-Anderson Respiratory Severity Score (RSS) Questionnaire	5/10	5/10	6/10	0/10	0/10	0/10
Oral Motor Assessment Scale	10/36	12/36	15/36	33/36	34/36	33/36

## Discussion

This case report underscores the triplets diagnosed with NRDS and low birth weight who had oromotor and respiratory complications. The clinical features of the condition include tachypnea, grunting, nasal flaring, retractions, decreased breath sounds, and poor feeding. Addressing the respiratory and oromotor complications in this vulnerable population is important for providing quality health care and reducing the further complications associated with this condition. To tackle the oromotor and respiratory complications of this condition, PROMPT protocol was administered.

 The PROMPT protocol is a systematically organized protocol comprising various physiotherapeutic techniques used in the betterment of feeding abilities, chest clearance, and overall neurodevelopment of a neonate diagnosed with neonatal respiratory distress syndrome. Oromotor stimulation includes multiple techniques like cheek stretch, upper lip stimulation, lower lip stimulation, upper gum stimulation, lower gum stimulation, tongue stimulation, and temporomandibular joint stroking, which are beneficial in improving feeding skills in preterm neonates. Manual airway clearance technique comprising of percussion, gentle vibrations, postural drainage, and reflex stimulation for respiratory facilitation helps in clearing the airway and improving the overall functions of the lungs. Swaddling positively impacts the neurodevelopment of neonates, helps in thermoregulation, enhances the motor development and feeding routine, and provides with a feeling of comfort and security. The tactile system is the first sensory system to develop in utero. Tactile stimulation is important for developing the bond between the mother and the child. It also plays a role in the neonate's neurodevelopment and weight gain. Kinesthetic stimulations help in the overall development of the neonate.

As noted by Atay et al., oromotor stimulation helps in improving the lip range of motion and lip seal, stimulates swallowing, and improves sucking. Oromotor stimulation helps in improving feeding skills in neonates [17]. It also facilitates an earlier transition to oral feeding and promotes early discharge of neonates. Gharu et al. stated that the reflex stimulation of respiratory facilitation improves breathing. Manual chest vibrations and percussion can promote improved lung function and airway clearance in infants with NRDS by removing mucus, increasing oxygenation, improving lung ventilation, and lowering respiratory workload and use of accessory muscles of respiration [18,19]. Ahmed and associates highlighted the value of kinesthetic and tactile stimulation as an all-encompassing approach to support the overall growth and well-being of neonates [20].

## Conclusions

The implementation of the PROMPT protocol in preterm neonates with NRDS showed significant improvement in their feeding ability and decreased respiratory complications, which indicates enhanced oromotor and respiratory capacities. Despite the encouraging results, larger cohort studies are required to confirm the effectiveness of the PROMPT methodology and to produce comprehensive, evidence-based research. In conclusion, for preterm triplets diagnosed with NRDS, the PROMPT protocol has demonstrated notable effectiveness in enhancing feeding abilities and reducing respiratory complications. These encouraging outcomes highlight the PROMPT protocol's potential as an efficient intervention for managing neonates with NRDS.
